# The Pandemic and the “Perpetual Foreigner”: How Threats Posed by the COVID-19 Pandemic Relate to Stereotyping of Asian Americans

**DOI:** 10.3389/fpsyg.2022.821891

**Published:** 2022-02-17

**Authors:** Jordan S. Daley, Natalie M. Gallagher, Galen V. Bodenhausen

**Affiliations:** ^1^Department of Psychology, Northwestern University, Evanston, IL, United States; ^2^Department of Psychology, Princeton University, Princeton, NJ, United States; ^3^Marketing Department, Kellogg School of Management, Evanston, IL, United States

**Keywords:** race, Asian Americans, stereotyping, COVID-19, pathogens, cultural foreignness

## Abstract

We examined the “othering” of Asian Americans in the context of the COVID-19 pandemic. Given past evidence that pathogen-related threat perceptions can exacerbate intergroup biases, as well as salient public narratives blaming the Chinese for the pandemic, we assessed whether individuals experiencing a greater sense of threat during the pandemic were more likely to apply the “perpetual foreigner” stereotype to Asian Americans. Over a seven-week period, we recruited 1,323 White Americans to complete a measure of the perceived Americanness of Asian, Black, and White targets. Asian targets were consistently perceived as less American than White targets, across variations in subjective health threat and regional case counts. The direct and indirect connections of political ideology to the observed patterns were examined, revealing that White participants who blamed China for the pandemic were more likely to apply the perpetual foreigner stereotype to Asian Americans. These results indicate that the othering of Asian Americans is pervasive among White Americans and that variables related to social conditions surrounding the COVID-19 pandemic can predict the potency of this association.

## Introduction

Stereotypes held about many social groups are rooted in historical patterns of intergroup relations, particularly patterns reflecting intergroup conflict and groups’ relative success and status (e.g., Fiske et al., 2007). Stereotype content often reflects perceptions of intergroup conflict, including both resource competition and conflicting cultural values (Bobo, 1988; Sears and Henry, 2003; for a review, see Bodenhausen and Richeson, 2010). Across time, new conditions emerge that can potentially modulate the content and expression of intergroup biases. For example, Whites’ expressions of ethnoracial bias have been tied to fluctuations in economic conditions (Bianchi et al., 2018) and to increases in the size of minority populations (Quillian, 1995; Craig and Richeson, 2014; Zou and Cheryan, 2021). Here, we examined whether the social upheaval associated with the COVID-19 pandemic and the patterns of perceived threat that it has given rise to are associated with changes in the expression of stereotypes about Asian Americans, given the origins of the pandemic in China.

Previous work in the United States has documented two dominant trends in the stereotyping of Asian people. First, they are often perceived as economically successful and admired as a “model minority” (Cheryan and Bodenhausen, 2020), though in fact people tend to substantially underestimate the economic gap between Asian Americans and Whites (Kuo et al., 2020). Second, they are also often viewed as foreign and as not representing cultural Americanness (Cheryan and Monin, 2005; Darling-Hammond et al., 2020). In fact, White Americans tend to perceive “Americanness” and “Whiteness” in very similar ways (Devos and Banaji, 2005; Devos and Heng, 2009), rendering non-White groups inherently less prototypically American. Thus, despite their presumed economic prowess, attributions of foreignness provide a basis for Asian Americans’ relative devaluation and subordination (Zou and Cheryan, 2017).

How might the COVID-19 pandemic influence this tendency toward “othering” Asian Americans? Chronic and manipulated pathogen threats have been linked to negative attitudes toward immigrants and people with unfamiliar backgrounds, and they amplify preferences for the (familiar) ingroup over the outgroup (Navarrete and Fessler, 2006; Green et al., 2010; Huang et al., 2011; Wu and Chang, 2012). Fear of uncertain health risks is linked to fear of unfamiliar (or supposedly *foreign*) others (e.g., Faulkner et al., 2004; Kusche and Barker, 2019; see Neuberg and Schaller, 2016). Thus, it is reasonable to hypothesize that pandemic-related fears will be linked to heightened othering of groups that are already viewed as relatively foreign. Anti-Asian sentiment and violence rose with the onset of the COVID-19 pandemic (e.g., Ruiz et al., 2020), especially when fear of the virus was high (Dhanani and Franz, 2020; Mandalaywala et al., 2020). While much of this prior work has emphasized broad, affective outcomes—whether unfamiliar groups are liked—we instead focus on the attribution of foreignness itself. Has COVID-19 pathogen threat impacted the application of the perpetual foreigner stereotype to Asian Americans?

Fears associated with the pandemic may be especially linked to Asians because this particular pathogen emerged in China. The origins of the pandemic in Wuhan suggest that Chinese people might be particularly subjected to heightened ethnoracial stereotyping by those threatened by COVID-19. However, many Americans fail to respect distinctions among different Asian ethnic groups; indeed, one of the most commonly reported microaggressions among Asian Americans is the failure of others to acknowledge ethnic group differences (Sue et al., 2007). To the extent that blame is focused on China, and Asian people more broadly (IPSOS, 2020), White Americans may be especially likely to view Asians as lacking “Americanness.” Media coverage of the pandemic, particularly the extent to which references to the virus implicate China (or Asian people more broadly), have been associated with heightened racial antipathies (Noel, 2020; Hswen et al., 2021), including controlled experimental research demonstrating a causal effect of emphasizing the COVID-China link (Dhanani and Franz, 2021). Additionally, supporters of then-President Donald Trump (who strongly emphasized the China-COVID-19 link; Rogers et al., 2020) reported more negative attitudes toward Asian people (Dhanani and Franz, 2020). These findings raise a key theoretical question—do perceptions of an outgroup’s foreignness rise as a function of perceived pathogen threat *per se*, or does there need to be an implied connection of the outgroup to the pathogen’s origins? Our study directly addresses this question.

## Materials and Methods

### Participants

During the summer of 2020, we recruited 1996 participants using TurkPrime’s CloudResearch platform (Litman et al., 2017). Recruitment took place over a seven-week period between June and August. This was a period when US COVID cases had surged again after an initial “flattening of the curve” and were at their highest 7-day average to date. The effects (socially, economically, and with regard to healthcare) of the pandemic were such that many thought that things in the United States might be at their worst stage. For example, US COVID-19 deaths surpassed 100,000, cases surpassed 2 million, and COVID-19 became the third-leading cause of death in the United States (AJMC, 2021).

One hundred and six participants were excluded: 16 said they had not taken the survey seriously, 8 reported different ages, states of residence, or political orientations across different attempts to participate, and 99 provided inauthentic open-ended text responses that were copied from another source; final *N* = 1,873. We used sampling quotas to balance representation across geographic and residential variables because of their relevance to the spread of COVID-19, and political ideology because of its psychological relevance to Americans’ individual beliefs about COVID-19 (Astor, 2021).

We report analyses with the subset of participants (70.05%, *N* = 1,312) who reported their racial/ethnic identity as monoracially White and who indicated their political ideology on the liberal-conservative continuum (60.90% men, 38.80% women, <1% other or no answer; Median education was a Bachelor’s degree (54.12%); *M*_age_ = 38.05; *SD*_age_ = 11.58). The sample was roughly balanced with respect to geographic region (23.48% in the Midwest, 20.73% in the Northeast, 35.00% in the South, and 20.81% in the West), residential population density (18.45% rural area, 27.82% small city or town, 31.56% suburb near a large city, 22.18% large city), and political ideology (43.60% liberal, 19.36% moderate, 37.04% conservative). We focused on White respondents because past research (e.g., Cheryan and Monin, 2005) suggests that the “othering” of Asian people is primarily found among White participants. Further, we restricted analyses to those who responded to a question about political ideology, given our intention to control for political ideology in the reported analyses, particularly the analyses that include the extent to which participants report holding China responsible for the pandemic.

### Measures

Through an online survey, participants completed a battery of measures assessing their beliefs and attitudes about different racial-ethnic groups residing in the United States. Here, we focus on two tasks assessing perceived Americanness—a semantic differential questionnaire (e.g., Osgood et al., 1957) and a face-rating task. These variables were studied as a function of participants’ characteristics and self-reported experiences during the COVID-19 pandemic in the summer of 2020.

#### Semantic Differential Measure

Participants were asked to think about *White people who live in the* United States and *Asian people who live in the* United States. For each group, participants choose from three 7-point scales with bipolar endpoints (American-Foreign, Familiar-Unfamiliar, Insider-Outsider). We collapsed across ratings and scaled this variable so that 1 is the most-American possible rating, and −1 is the most-Foreign possible rating (*α*_Asian_ = .87; *α*_White_ = .9).

#### Face-Rating Task

Participants also completed a face-rating task modeled after Study 1 of Cheryan and Monin (2005). Participants rated target faces for how American, Attractive, and Intelligent they were, with the latter two being fillers. Ratings were collected using a slider ranging from *Not at all/0* to *Extremely/100*; for analysis, the Americanness ratings were rescaled as a −1 to 1 variable, where 1 is Extremely American. Ratings were made for two faces each from eight groups defined by: perceived race (Asian vs. White), perceived gender (man vs. woman), and supposed birthplace of origin (United States vs. non-United States).^1^ To use naturalistic faces for the rating task, we chose faces from the 10 k face database (Bainbridge et al., 2013), using a combination of the norming data provided by the authors and norming data that we gathered using the same process and population as our main data collection (*N* = 170; *M*_age_ = 38.42 years, *SD*_age_ = 11.78; 61.20% men, 38.2% women, .59% not listed; 77.1% White, 11.8% Asian, 8.8% Black, 6.5% Hispanic/Latinx, 2.9% Native American, .6% Pacific Islander). Each face was categorized as a member of a particular racial-ethnic or gender group by at least 90% of norming participants, as well as being judged to be happy, between the ages of 20 and 60, of good image quality, and looking toward the camera. We then used propensity matching (Randolph and Falbe, 2014) to select two sets of faces in which the stimulus groups (defined by perceived race and perceived gender, e.g., Asian women) were matched on apparent age, attractiveness, friendliness, image quality, and whether the image would be a good profile picture. This image selection process was intended to provide sets of images that differed by race and gender but were otherwise quite similar in their content and quality. Using two sets of matched images allows for greater generalization across specific faces.

Participants were randomly assigned to the first or second set of faces. Within that set of faces, we used the names of small towns to list an ostensible birthplace for each target. Asian targets were pseudo-randomly paired with a birthplace either in the United States or in China, while White targets were pseudo-randomly paired with a birthplace either in the United States or in the UK.

#### COVID Self-Report

Our analyses focus on responses to two questions related to participants’ experiences during the pandemic. Participants reported their perceived risk of contracting COVID-19 (“What is your risk of contagion from COVID-19?”; *1/Much lower risk than the average US resident*—*7/Much higher risk than the average US resident*; *M* = 4.35, *SD* = 1.42) and the extent to which they hold China responsible for the pandemic (“Do you think China is responsible for the global COVID-19 pandemic?”; *1/Not at all responsible*—*7/Entirely responsible*; *M* = 4.97, *SD* = 1.73). We use the responses to these items as two central predictor variables.

#### Actual COVID Rates

We used The New York Times (2021) COVID database and 2019 US census population projections to calculate the one-week rolling average of new cases *per capita* in each US state during our period of recruitment. We matched this with participants according to their state of residence. Across our data, this metric ranges from .009 to .552%. This means that, during the prior 7 days, there were 9–552 new cases per day per 1,000,000 people in each participant’s state. We use the average across a week to avoid effects of days of the week (e.g., higher reporting on Tuesdays as weekend counts were integrated). For analysis purposes, we rescaled this variable to go from 0 to 1.

## Results

We were centrally interested in whether White participants’ exposure to COVID-19 would predict judging Asian people as less American. We examined the extent to which Americanness ratings are predicted by actual COVID rates in the participant’s state, the participant’s subjective sense of health risk from COVID, the extent to which the participant held China responsible for the pandemic, and participant political ideology. We present results from the semantic differential and face-rating tasks separately, using a single simultaneous regression model for each dependent variable with follow-up tests as described. Thus, all results reflect the unique effect of a particular predictor above and beyond the predictor’s shared variance with other predictors (see Table 1 for correlations between predictors).

**Table 1:** Correlations between predictor variables.

S. no.	Variable	1	2	3
1.	COVID rates in state	–		
2.	Subjective health risk	.043	–	
3.	Political Ideology	.085^**^	.094^***^	–
4.	Holding China Responsible	.093^***^	.280^***^	.348^***^

** *p* < .01;

*** *p* < .001.

We use a multi-level modeling approach to account for participant-level differences in overall tendency to rate groups or individuals as more or less American (Raudenbush and Bryk, 2002; Bates et al., 2015). We report significance for fixed effects using Satterthwaite’s method (Kuznetsova et al., 2017), and all reported means are estimated marginal means. Our code and a de-identified data file can be found on OSF at: https://osf.io/b3wu6/. Robustness checks, including analyses run with the full sample (no participant exclusions) and run with data quality exclusions, but no race constraints, indicate that our central findings hold regardless of these exclusions. All of these datasets are available at the OSF link above.

### Semantic Differential

To see whether COVID variables predict semantic differential ratings, we examined perceived Americanness as a function of target group (Asian-White), two COVID-related individual difference variables (actual COVID risk in state, subjective health risk), two political individual difference variables (blaming China for the pandemic, political ideology), and the interactions of these individual difference variables with target group. Key questions concern (a) whether we replicate the “perpetual foreigner” stereotype in which Asian people are judged less American than White people, and (b) whether the size of that effect differs according to any of our predictors. We account for the repeated-measures nature of the data by including a random intercept of participant.

**Figure 1:**
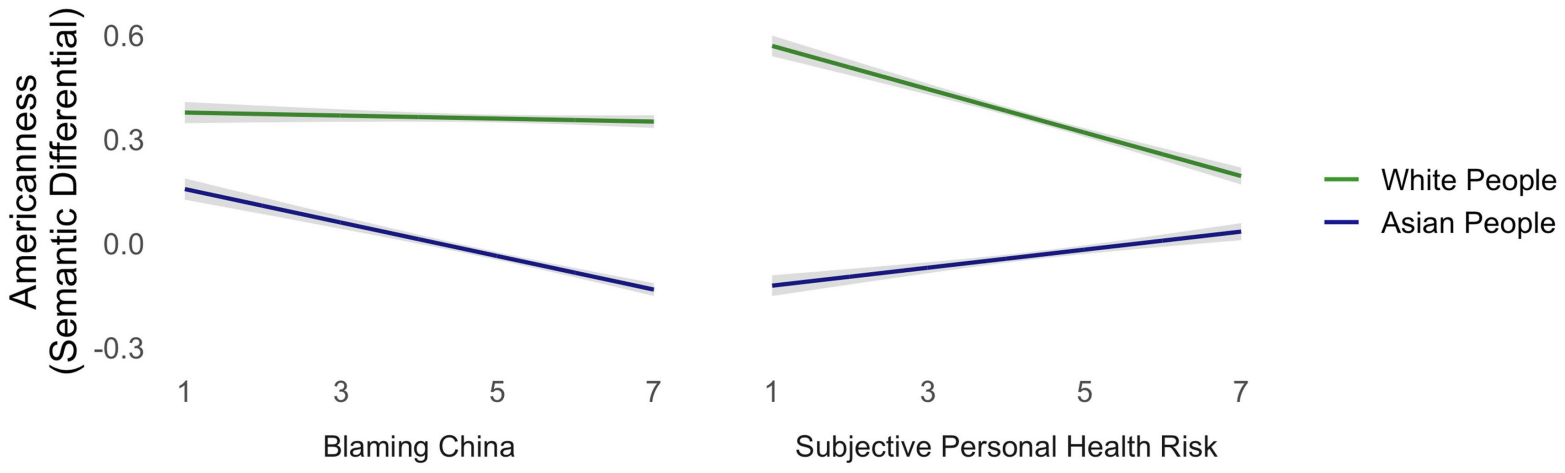
Participants with greater subjective health risk rated Asian faces—and especially Asian faces associated with US birthplaces—more American than did participants lower in subjective health risk.

There was a main effect of target race/ethnicity, such that White people living in the United States were rated more American than Asian people living in the United States [*t*(1291) = 10.623, *p* < .0001; *M*_Asian_ = −.0343, *SE*_Asian_ = .01, *M*_White_ = .3598, *SE*_White_ = .01]. Neither actual state-level COVID rates nor political ideology predicted overall foreignness ratings or differential foreignness ratings by target group (*p*s *>* .2). Sense of personal health risk and blaming China for the pandemic predicted lower Americanness ratings overall [personal health risk: *t*(1292) = −2.754, *p* = .006; blaming China: *t*(1300) = −4.563, *p* < .0001]. The effects of blaming China and subjective health risk both differed by target group [personal health risk: *t*(1291) = −9.060, *p* < .0001; blaming China: *t*(1288) = 5.184, *p* < .0001]. While blaming China predicted rating Asian people (but not White people) as less American, subjective health risk predicted rating White people as less American and Asian people as more American (see Figure 1).

### Face Evaluation

We used a similar modeling approach to look at each participant’s 16 target face Americanness ratings. We include a random intercept for specific target face. We also include a fixed effect of birthplace (United States vs. outside of the United States), a fixed effect for the interaction of birthplace and target group, and fixed effects for these interacting with our individual difference predictors.

Once again, White faces were rated as more American than Asian faces [*t*(278) = 20.014, *p* < .0001]. Faces associated with a US birthplace were rated as more American than those associated with a non-US birthplace [*t*(19520) = 16.708, *p* < .0001]. These two effects interacted, such that the birthplace “penalty” was greater for White people than Asian people [*t*(19520) = 2.953, *p* = .003; Asian faces: *t*(9105) = 10.934, *p* < .0001; White faces: *t*(9119) = 15.779, *p* < .0001].

Once again, actual COVID cases did not predict overall attributions of Americanness (*p* = .45). Actual COVID cases also did not predict differential attribution of Americanness based on birthplace (*p* = .18), or by the interaction of birthplace and target group (*p* = .29). However, it did predict differential attribution of Americanness by target group [*t*(19520) = −4.727, *p* < .0001], such that it predicted rating White people (but not Asian people) as slightly less American [White faces: *t*(1300) = −2.487, *p* = .01; Asian faces: *p* > .45].

Further, political ideology did not predict overall attributions of Americanness (*p* > .63). It predicted differential attribution of Americanness by target group [*t*(19520) = 4.495, *p* < .0001] and birthplace [*t*(19520) = −5.512, *p* < .0001], but not by the interaction of group and birthplace (*p* > .85). The Asian/White difference in Americanness was larger among conservatives than liberals, while the US-born/Foreign-born difference in Americanness was smaller among conservatives than liberals.

**Figure 2:**
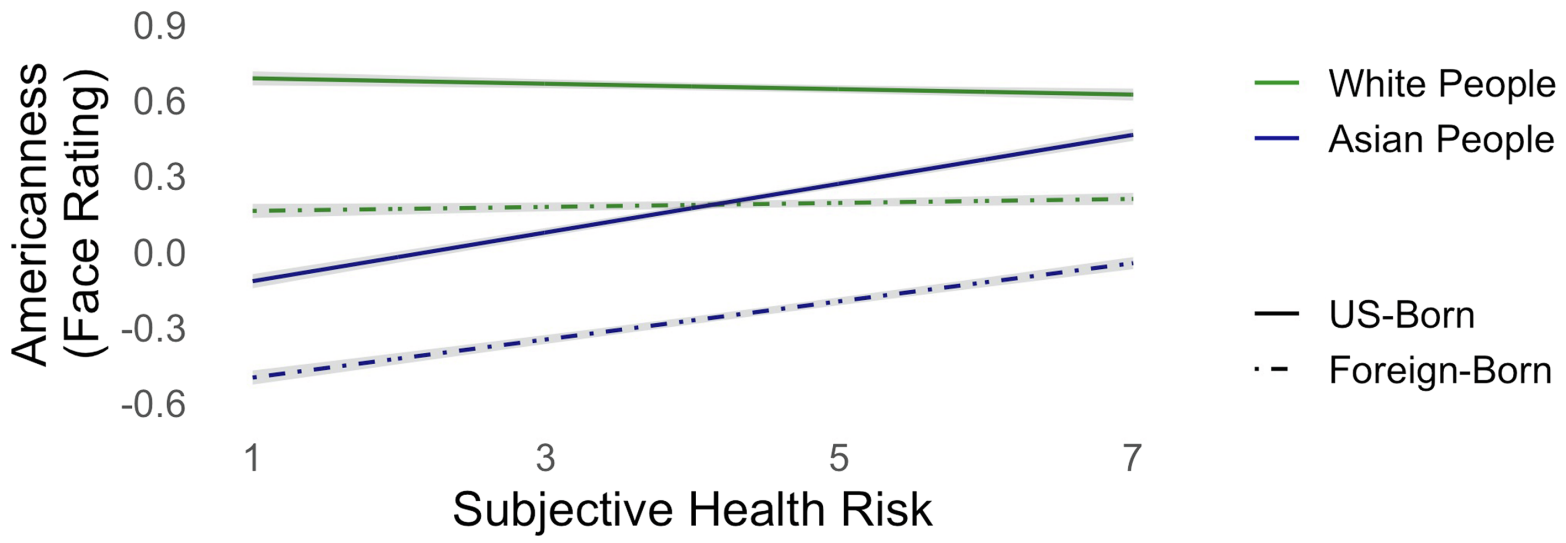
Blaming the COVID-19 pandemic on China predicted greater Asian-White differentiation in attributing Americanness and greater differentiation on birthplace in attributing Americanness.

Subjective health risk predicted rating faces as more American overall [*t*(1301) = 7.267, *p* < .0001]. It also predicted differential rating by target group [*t*(19530) = −18.965, *p* < .0001] such that subjective health risk predicted rating Asian faces as significantly more American [*t*(1301) = 10.051, *p* < .0001], but had no significant effect on ratings of White faces (*p* > .8). Though it did not predict differential rating by birthplace (*p* > .79), it did predict differential rating based on the interaction of birthplace and target group [*t*(19530) = −4.304, *p* < .0001]. Altogether, this means that participants reporting greater sense of health risk rated Asian (but not White) faces as more American and that this effect was greatest for Asians associated with US birthplaces (see Figure 2).

**Figure 3:**
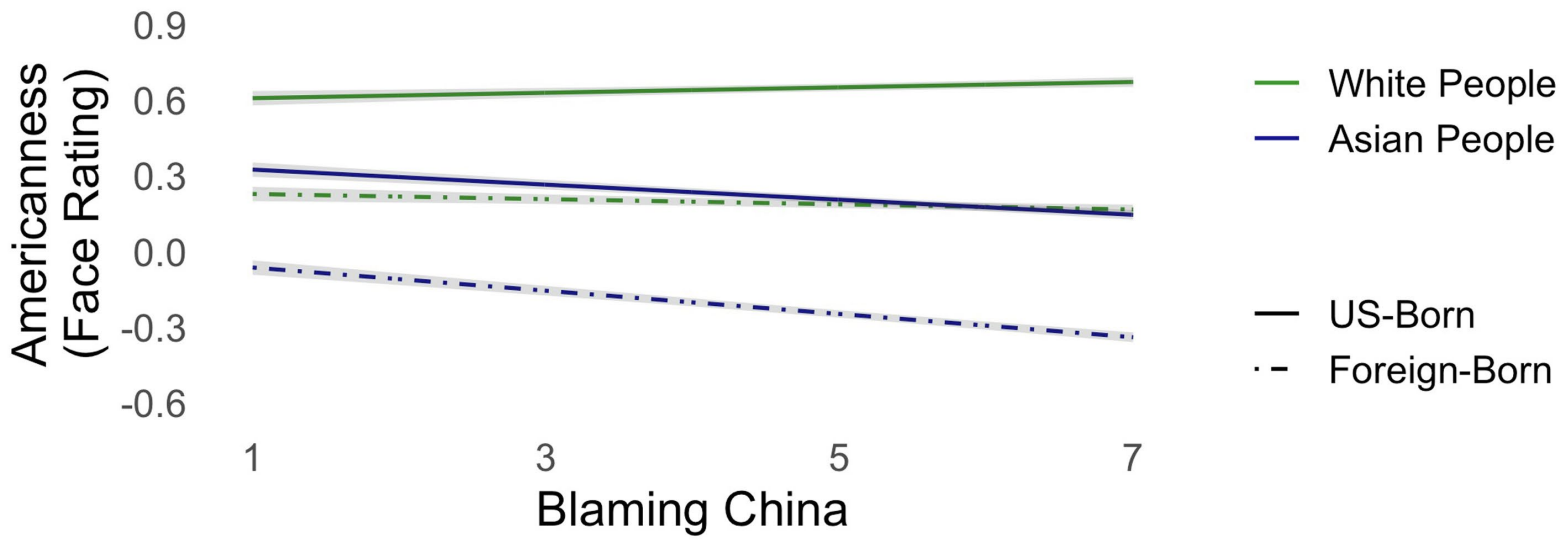
Blaming China for COVID predicted rating Asian people as less American, while sense of health risk predicted rating White people as less American and Asian people as more American.

Blaming China predicted rating faces as less American overall [*t*(1300) = −3.722, *p* < .001]. It also predicted differential ratings by target group [*t*(19520) = 9.516, *p* < .0001] and by birthplace [*t*(19520) = 4.6128, *p* < .0001], but not by the interaction of target group and birthplace (*p* > .55). Blaming China for the pandemic predicted greater Asian-White differentiation in Americanness ratings, and greater US-born/Foreign-born differentiation in Americanness ratings (see Figure 3).

## Discussion

These findings provide a snapshot of White Americans’ experiences during the first summer of the COVID-19 pandemic and how these experiences are connected to perceptions of Asian people in America. Specifically, we probed the potency of the “perpetual foreigner” stereotype of Asian Americans among White Americans (Cheryan and Monin, 2005) during an unprecedented international public health crisis. While previous work has focused on the relationship between pathogen concern and increased levels of prejudice toward certain groups, we focus instead on the attribution of Americanness (versus foreignness) to Asian targets. In particular, we explored whether attributions of Americanness were associated with pathogen threat *per se* or other sociopolitical attitudes related to this threat, namely, the extent to which people blame China for the pandemic.

Consistent with prior research, our participants rated White people as significantly more American than Asian people on both a composite semantic differential scale (American-Foreign, Familiar-Unfamiliar, Insider-Outsider) and a face-rating measure. We were particularly interested in exploring how this tendency might intersect with attitudes and experiences related to the COVID-19 pandemic. In contrast with expectations based on previous literature, neither actual COVID-19 case rates nor subjective health risk predicted lower Americanness ratings for Asian targets. In fact, although previous literature would suggest that increased pathogen threat due to the pandemic is likely to *negatively* influence intergroup perceptions (Dhanani and Franz, 2020; Mandalaywala et al., 2020), we find evidence for the opposite effect in our sample. That is, when a participant’s health concern was higher, the Asian target group and Asian faces were judged as more American than when subjective health concern was lower.

Interestingly, we find that the gap in the perceived Americanness of White versus Asian people narrows as the perceived risks from COVID-19 increase, not only due to increased perceptions of the Americanness of Asian people, but also because of decreased perceptions of the Americanness of White people. Although this finding seems inconsistent with the idea that pathogen threat promotes intergroup biases, the COVID-19 context may be unusual in that a number of influential public voices argued that the virus was actually not a serious danger (Imhoff and Lamberty, 2020; Lewis, 2020). Subjective feelings of threat thus require perceiving the actual pathogen-related risk despite salient attempts to trivialize or minimize it. Weil and Wolfe (2021) have shown that perceived risks from COVID, and intentions to take self-protective actions, are positively correlated with greater cognitive reflection. Greater cognitive reflection, in turn, predicts a reduced tendency to think in simplistic, stereotypic ways and reduced expression of racial biases (e.g., Waller, 1993; Toplak et al., 2011). Conversely, feeling relatively invulnerable to a pathogen can reflect a tendency toward ego-defensive cognitive styles (Harris et al., 2008), which have also been associated with a greater need to view ingroups more favorably than outgroups (e.g., Allport, 1954; Hepper et al., 2010). Thus, White Americans who report higher personal vulnerability to COVID-19 may have a lower likelihood of thinking about racial groups in stereotypic ways because of broader cognitive tendencies (i.e., non-defensive cognitive reflection) that go along with recognizing and acknowledging the risks posed by COVID-19.

Sociopolitical attitudes proved to be relevant predictors of the magnitude of the perpetual foreigner bias. Although both liberal and conservative participants stereotyped Asians as relatively foreign, the effect was larger among conservative participants (at least when rating faces). Moreover, increasing tendencies to blame China for the pandemic were associated with viewing Asian people as more foreign. Scholars have suggested that discourse around the naming of a virus and the source of a virus can have implications for intergroup attitudes and relations (Hoppe, 2018; Cho et al., 2021). Our results seem to support these claims, and in fact, we find that blaming China for the pandemic was a stronger predictor of negative intergroup perceptions than health threat.

In our data, neither actual COVID-19 case rates nor reported subjective health risk predicted heightened associations between Asians and foreignness. It may be that this stereotypic association operates differently from general prejudice, in terms of how White Americans respond to a pathogen threat. Although the threat engendered by the COVID-19 pandemic has been linked with increases in anti-Asian sentiment and violence, our data suggest that perceptions of foreignness may not be the psychological mechanism behind this discrimination, as subjective pathogen threat was associated with reductions in foreignness attributions. However, it may be that a small subset of the population does react to pathogen threat with increased perceptions of foreignness toward Asian Americans and that the documented offenses are driven by this atypical, “concentrated” group (Campbell and Brauer, 2021).

Even so, we do find evidence consistent with the notion that Asians are perpetually perceived as foreign, relative to White people, and that increased blame for the pandemic directed toward China is associated with increased attributions of foreignness toward Asian people. Biased attributions of foreignness to groups of Americans are a manifestation of ongoing xenophobia and ethnonationalism in modern society. As such, it has immediate and everyday impacts on the lives of people culturally deemed “un-American.” Presumptions of foreignness directed toward Asian Americans constitute an everyday microaggression (Sue et al., 2007, 2009) that can decrease feelings of belonging (Huynh et al., 2011) and negatively impact mental health (e.g., affect, stress, and depressive symptoms; Wang et al., 2013; Albuja et al., 2019a,b). Similar results have been shown in other racial-ethnic groups culturally stereotyped as “foreign” (e.g., Barlow et al., 2000; Sanchez et al., 2018). Biased perceptions of foreignness can thus have substantial adverse consequences for both individuals and groups.

### Limitations

Our study has several limitations. First, all our effects should be considered associative, rather than causal, as we do not manipulate any of our predictor variables. Further, as outlined in our methods, we constrain our analyses to focus on just White American participants. Thus, our results should not be assumed to generalize beyond this sub-population. In addition, although previous work suggests that many White Americans blur distinctions between different Asian cultural and national groups, there are clear reasons to believe that studying perceptions of specific Asian subgroups could yield meaningful nuance (Goh and McCue, 2021). In our study, we followed prior work in focusing on Asian Americans as an aggregate group, but we recognize that investigations focusing on perceptions of Asians with different national heritages could prove extremely valuable. Finally, we acknowledge that respondents’ considered responses to an online survey may not necessarily be indicative of their spontaneous behavior in actual daily life contexts, including intergroup contact situations.

## Conclusion

In a robust investigation of White Americans’ perceptions of Asian and Asian American people, we find evidence of the “perceptual foreigner” stereotype of Asians in America. In addition, we find that COVID-19 case rates and subjective health risk during the pandemic do not predict increased attributions of foreignness toward Asian people. However, we do find that sociopolitical attitudes, such as assigning blame to China for the pandemic do predict increased foreignness perceptions of Asians. Together, we provide an interesting complement to previous literature focusing on the effects of pathogen concern and intergroup attitudes.

## Data Availability Statement

The original contributions presented in the study are publicly available. This data can be found here: https://osf.io/b3wu6/.

## Ethics Statement

The studies involving human participants were reviewed and approved by Northwestern University Institutional Review Board. The participants provided their express informed consent to participate in this study.

## Author Contributions

JD, NG, and GB contributed to conception and design of the study and wrote sections of the manuscript. JD and NG both prepared and administered the survey tool for data collection and organized and cleaned the database(s). NG performed most of the statistical analysis and led the efforts for the acquisition of funding. JD performed supplemental analyses, including robustness checks and wrote the first draft of the manuscript. All authors contributed to manuscript revision, read, and approved the submitted version.

## Funding

This project was funded by Northwestern University’s Weinberg College COVID-19 Research Seed Fund Program.

## Conflict of Interest

The authors declare that the research was conducted in the absence of any commercial or financial relationships that could be construed as a potential conflict of interest.

## Publisher’s Note

All claims expressed in this article are solely those of the authors and do not necessarily represent those of their affiliated organizations, or those of the publisher, the editors and the reviewers. Any product that may be evaluated in this article, or claim that may be made by its manufacturer, is not guaranteed or endorsed by the publisher.

## Footnotes

^1^Participants also evaluated the faces of four Black people assigned to US birthplaces; these ancillary data are omitted from the present analysis.
